# Burst wave lithotripsy - a paradigm shift: inferences from a scoping review

**DOI:** 10.1007/s00345-025-05645-x

**Published:** 2025-04-25

**Authors:** Steffi Kar Kei Yuen, Vineet Gauhar, Chu Ann Chai, Connor M. Forbes, Victor K. F. Wong, Ryan F. Paterson, Ivan Ching Ho Ko, Joseph Li, Daniele Castellani, Ben H. Chew

**Affiliations:** 1 S. H. Ho Urology Centre, Department of Surgery, The Chinese University of Hong Kong, Hong Kong, China; 2 European Association of Urology Section of Endourology (ESEUT), Arnhem, The Netherlands; 3 Department of Urology, Ng Teng Fong Hospital, Singapore, Singapore; 4 Department of Surgery, Urology Unit, University of Malaya, Kuala Lumpur, Malaysia; 5 Department of Urology, University of British Columbia, Vancouver, Canada; 6 Department of Surgery, Tuen Mun Hospital, Hong Kong, China; 7 Urology Unit, Azienda Ospedaliero Universitaria delle Marche, Ancona, Italy

**Keywords:** Lithotripsy, Break wave, Burst wave, Nephrocalcinosis, Ultrasound propulsion, Shock wave

## Abstract

**Purpose:**

Urolithiasis, a condition affecting approximately 10% of the global population, is primarily treated with Shock Wave Lithotripsy (SWL) and endoscopic methods. However, SWL’s high-pressure pulses can cause tissue injury, often necessitates some level of anaesthesia, and may require repeated sessions or ancillary treatment to achieve stone free status. Burst Wave Lithotripsy (BWL) emerges as a promising alternative, utilizing multi-cycle, ultrasound bursts at lower pressure amplitude to fragment stones, while minimizing cavitation under real-time imaging in a portable manner.

**Methods:**

This scoping review evaluated BWL’s efficacy, safety, and clinical potential. A systematic search identified 19 eligible studies, including in vitro experiments, preclinical trials, and human clinical trials.

**Results:**

In vitro studies demonstrated BWL’s capability to fragment urinary stones of diverse compositions with high comminution rates. Higher ultrasound frequencies produced smaller fragments (< 1 mm), while lower frequencies resulted in larger fragments (3–4 mm), allowing for controlled fragmentation tailored to clinical needs. Preclinical trials in porcine models showed lower pressure and reduced cavitation, which account for BWL’s safety, causing less associated tissue injury even in anticoagulated subjects. Human trials reported BWL as well tolerated in awake patients with high fragmentation success rates (88–91%) and low complication rates. BWL offers distinct advantages, including lower cavitation and tissue injury risks, portability, and anaesthesia-free application. Ongoing trials aim to validate BWL’s efficacy and explore its combined use with ultrasonic propulsion.

**Conclusion:**

BWL represents a paradigm shift in lithotripsy, offering controlled fragmentation, reduced tissue injury, fragment propulsion and portability for office-based or ambulatory care. Early clinical evidence underscores its safety and efficacy, even in anticoagulated patients. While large-scale trials are needed to solidify its role, BWL’s procedural flexibility positions it as a transformative alternative to SWL, poised to redefine urolithiasis management.

**Supplementary Information:**

The online version contains supplementary material available at 10.1007/s00345-025-05645-x.

## Introduction

Urolithiasis represents a significant challenge for urologists due to its high prevalence, which is estimated to be around 10% globally [1]. Common treatment options include Shock Wave Lithotripsy (SWL) and endoscopic lithotripsy, with the choice depending on the size and location of the stone. SWL delivers single-cycle pulses of energy at a slow rate (≤ 2 Hz) and high peak pressures [2, 3], which often necessitates some level of anesthesia. Shock-induced cavitation at the stone surface can initiate fractures [4]. However, the proliferation of cavitation clouds caused by delivering shocks at too high a rate reduces their effectiveness and may lead to tissue damage [5]. Burst wave lithotripsy (BWL), a new technology capable of fragmenting stones without requiring anesthesia or sedation with minimal pain and complications, has been gaining traction.

**Fig. 1:**
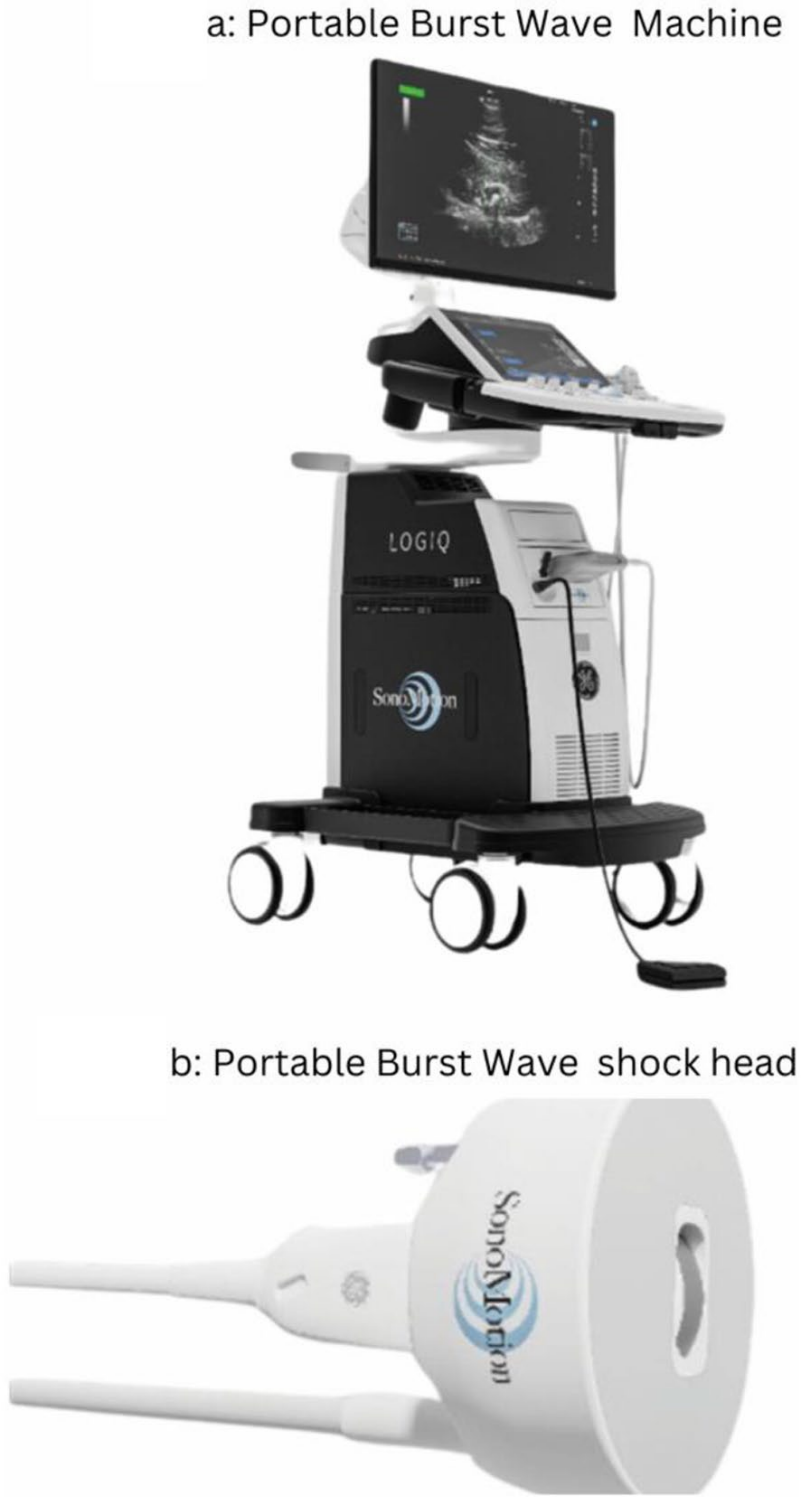
(**a**) Burst Wave Lithotripsy machine; (**b**) Burst Wave Lithotripsy probe

The BWL system consists of a small therapy transducer that is applied to the skin with ultrasound gel and a diagnostic ultrasound imaging system with an integrated electronic pulser. The entire system is portable and can be mounted on a single user-friendly trolley. (Fig. 1A) BWL employs multi-cycle focused ultrasound pulses (Fig. 1B) with a beam width equal to or greater than the stone to excite elastic waves within the stone, producing standing stress waves within the stone resulting in discrete fragments rather than dust-like particles [6]. Unlike SWL, BWL uses sinusoidal ultrasound bursts characterized by relatively low-pressure amplitudes, which effectively minimizes the accumulation of cavitation bubbles. Real-time diagnostic ultrasound imaging provides continuous monitoring of the procedure, which allows for both proper stone targeting and identification and mitigation of cavitation in the event it occurs. This reduction in cavitation enhances acoustic waves propagation into the stone, thereby increasing the likelihood of successful stone fracture [5] and reduces the likelihood of adverse events caused by cavitation. A potential advantage of BWL over SWL is its ability to produce smaller fragment sizes [6] without any anesthesia, on a fully awake patient at any medical setting (Table 1).

Utilizing the same probe as BWL system, ultrasonic propulsion technology to reposition stones can be delivered transcutaneously with real-time ultrasound image guidance and targeting. Ultrasound propulsion pulses have longer duration and lower amplitude than BWL pulses.

Over the past nine years, this technology has progressed from concept to human clinical trials [7-9]. In this review, we analyze current evidence for burst wave lithotripsy and explore its potential future applications. Our primary aim in this scoping review is to highlight how experimental evidence is now being validated in human clinical studies and to postulate the main advantages this technology is poised to offer.

**Table 1:**
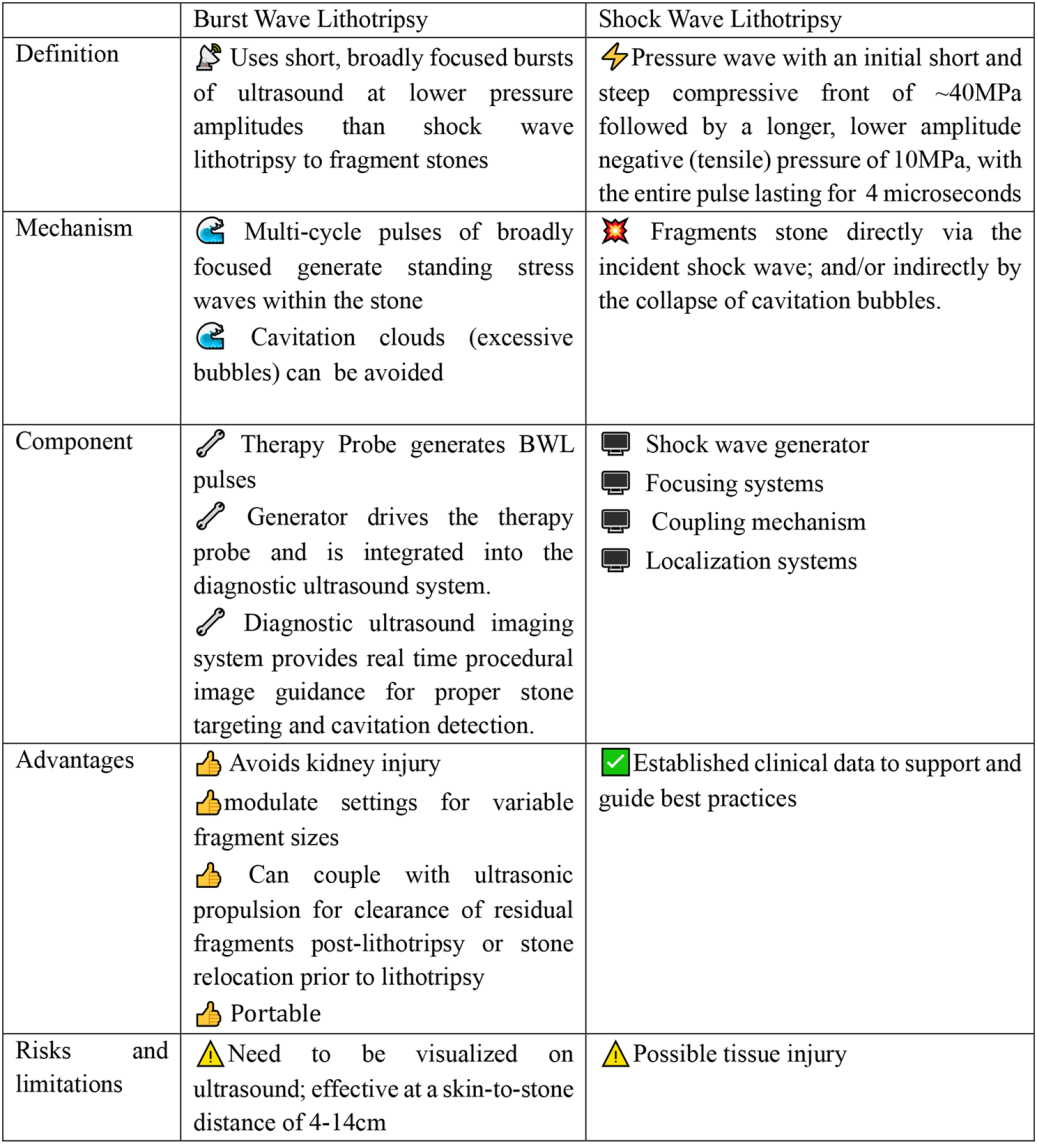
Comparing burst wave lithotripsy to shock wave lithotripsy

### Material and method

The publication search was conducted from inception to 21 January 2025 in several databases, including the Embase, PubMed, Scopus, and the Cochrane Central database via Boolean operators with use of following terms: (“burst wave " OR “break wave " OR “Propulsion”) AND (“kidney " OR “renal " OR “ureter " OR “ureteral “) AND (“stone” OR “calculi” OR “urolithiasis”) to identify relevant studies on Burst Wave Lithotripsy. Search restrictions were set for English publication. Duplicates were removed from screening. We included all clinical and experimental studies that evaluate BWL. Non-english articles, abstracts and editorials were excluded. The final selection of literature analysis was confirmed by two independent authors. This scoping review was performed in accordance with the Preferred Reporting Items for Systematic reviews and Meta-Analyses (PRISMA) guidelines [10].

## Results

A total of 160 studies were screened against title and abstract. 52 studies assessed for full-text eligibility, in which 33 studies excluded. After the selection process, 19 records met the eligibility criteria and were included in this scoping review (Supplementary Figure). Table 2 summarizes the characteristic of the included studies.

**Table 2:** Characteristics of the included studies

Author	Published Year	Type of Study	Aim of study
Maxwell, et al. [6]	2015	In vitro	To evaluate the in vitro fragmentation of urinary calculi by BWL
May, et al. [24]	2017	In vitro	Detection and Evaluation of Renal Injury in BWL
Zwaschka, et al. [14]	2018	In vitro	To evaluate the outcome of combining BWL and ultrasonic propulsion for stone fragmentation
Maxwell, et al. [21]	2019	Animal	Evaluation of renal stone comminution and injury by BWL in a porcine model
Wang, et al. [18]	2019	Animal	To evaluate the efficacy and safety of BWL using a porcine model
Moghimnezhad, et al. [11]	2020	In vitro	To evaluate BWL, synthesis of Shock and Burst Waves
Ramesh, et al. [13]	2020	In vitro	In vitro evaluation of BWL system
Maxwell, et al. [16]	2020	In vitro	To evaluate stress patterns in model stones and study wave propagation
Maxwell, et al. [17]	2021	In vitro	To evaluate factors affecting tissue cavitation during BWL
Harper, et al. [25]	2021	Human	To evaluate BWL for kidney stone comminution in human
Sapozhnikov, et al. [15]	2021	In vitro	To develop an analytical model, validate it against a numerical (finite difference) model, and then use both to investigate the effect of frequency on stress magnitude in stones exposed to BWL pulses
Connors, et al. [22]	2022	Animal	To determine if clinical BWL exposure produces a functional or morphological change in the kidney
Bailey, et al. [12]	2022	In vitro	To investigate how to fragment small stones and why varying the BWL frequency may more effectively fragment stones to dust
Hall, et al. [26]	2022	Human	To evaluate ultrasonic propulsion and BWL in treatment of ureteral stones
Harper, et al. [7]	2022	Human	To report the outcome of BWL of urinary stones in first 19 human
Maxwell, et al. [19]	2023	Animal	Development of a BWL system for pet cats
Chew, et al. [8]	2024	Human	To report the outcome of first in-human international multi-institutional clinical trial for BWL
Holmes, et al. [20]	2024	Animal	To report the outcome of novel BWL and ultrasonic propulsion technology for the treatment of ureteral stone in a bottlenose dolphin (Tursiops truncatus) and renal calculi in a harbor seal (Phoca vitulina)
Shelton, et al. [23]	2025	Animal	To evaluate if BWL could be used in pigs undergoing anticoagulation therapy
Maxwell, et al. [6]	In vitro	Artificial and natural stones (mean±SD size 8.2± 3.0 mm, 5 to 15 mm)	Largest stone fragments produced from ultrasound frequencies exposure of 170, 285 and 800 kHz were less than 4 mm, less than 2 mm and less than 1 mm, respectively.
May, et al. [24]	In vitro	Ten kidneys from five anesthetized female pigs	Observed B-mode hyperechoes on ultrasound consistent with cavitation predicted the presence of BWL-induced renal injury with a sensitivity and specificity of 100% in comparison to the histomorphometric technique. MRI detected renal injury with a sensitivity of 90% and specificity of 100%.
Zwaschka, et al. [14]	In vitro	Artificial BegoStone and human calcium oxalate monohydrate 5 to 8 mm	BWL with ultrasonic propulsion improved stone fragmentation compared with BWL alone in vitro, from 6–11% (*p* < 0.001) in BegoStone model comminution and from 17–36% (*p* = 0.01) with interleaved propulsion in calcium oxalate monohydrate stones.
Maxwell, et al. [21]	Animal	6 to 7 mm human kidney stone was surgically implanted in each kidney of three pigs	On average, 87% of the stone mass was reduced to fragments < 2 mm. No injury was detected through gross, histologic, or MRI examination in the parenchymal tissue.
Wang, et al. [18]	Animal	6 to 7 mm human kidney stone was surgically implanted in each kidney of three pigs	An average 87% or 92% of the mass was comminuted to fragments < 2 mm or < 4 mm respectively. Evaluation by MRI did not detect hemorrhagic injury to the functional renal volume.
Moghimnezhad, et al. [11]	In vitro	Not applicable	Increasing frequency of BWL leads to a reduction of the focal region, which reduced damages to adjacent tissues. Stone material has a key role in the distribution and amount of von Mises stress in the stones. Thermal analysis shows that the temperature increases only inside the stone, and none to surrounding tissues.
Ramesh, et al. [13]	In vitro	46 human stones, each 5–7 mm	89% (41/46) and 70% (32/46) of human stones were fully comminuted within 30 and 10 min, respectively.
Maxwell, et al. [16]	In vitro	Not applicable	Photoelasticity imaging captured by high-speed camera showed the development of periodic stresses in the stone body with a pattern dependent on frequency.
Maxwell, et al. [17]	In vitro	Kidneys of eight pigs	Lowering pulse repetition frequency was found to significantly increase the pressure amplitude needed to produce sustained cavitation, and controlling this parameter may offer a mechanism to avoid sustained cavitation and renal injury during BWL procedures.
Harper, et al. [25]	Human	2 human subjects	In one participant, a ureteroscope inserted after 9 min of BWL observed fragmentation of the stone to < 2 mm fragments; whilst the other participant tolerated the procedure without pain from BWL, required no anesthesia, and passed the stone on day 15.
Sapozhnikov, et al. [15]	In vitro	Not applicable	Results from the analytical model suggest that BWL frequency should be elevated for small stones to improve the likelihood and rate of fragmentation.
Connors, et al. [22]	Animal	Twelve female pigs	No visible gross hematuria was observed in any of the collected urine samples of the treated kidneys. BWL exposure also did not lead to a change in GFR after treatment, nor did it cause a measurable amount of hemorrhage in the tissue.
Bailey, et al. [12]	In vitro	Matched pairs of stones ranging 1–5 mm	The threshold frequency for stress amplification is proportionate to the wave speed divided by the stone diameter. Increasing frequency of BWL may produce amplified stress in the stone causing the stone to break.
Hall, et al. [26]	Human	29 human subjects	This study supports the efficacy and safety of using ultrasonic propulsion and burst wave lithotripsy in awake subjects to reposition and break ureteral stones to relieve pain and facilitate passage.
Harper, et al. [7]	Human	19 human subjects	This was a prospective multi-institutional feasibility study, demonstrating a median of 90% comminution of the total stone volume into fragments 2 mm within 10 min of BWL exposure with only mild tissue injury.
Maxwell, et al. [19]	Animal	35 calcium oxalate monohydrate stones from cats	The experiment demonstrated an average of 73–97% of stone mass reduced to fragments < 1 mm with BWL, supporting it as a noninvasive intervention for obstructing stones in cats.
Chew, et al. [8]	Human	44 human subjects	BWL offered safe and effective noninvasive stone therapy, requiring little to no anesthesia and was carried out successfully in nonoperative environments. Stone fragmentation occurred in 88%, 70% had fragments 4 and 51% 2 mm, while 49% were completely stone free on CT; no serious adverse events were reported.
Holmes, et al. [20]	Animal	A 48-year-old female bottlenose dolphin (*Tursiops truncatus*) and a 23-year-old male harbor seal (*Phoca vitulina*)	BWL and ultrasonic propulsion successfully relieved ureteral stone obstruction in a geriatric dolphin and reduced renal stone burden in a geriatric harbor seal.
Shelton, et al. [23]	Animal	Six pigs	A typical clinical dose of BWL (18,000 ultrasound pulses at 10 Hz, 20 cycles/pulse, peak positive pressure of 12 MPa) did not cause any hemorrhagic injury to the kidney even during therapeutic anticoagulation therapy, suggesting that BWL should be safe to use in patients with stone undergoing anticoagulation/antiplatelet therapy.

BWL– Burst Wave Lithotripsy; SD– standard deviation

## Discussion

### In vitro evidence

In vitro experiments on the effectiveness of BWL across multiple studies demonstrate its potential as a noninvasive, effective, and controlled method for fragmenting urinary stones of various compositions, including calcium oxalate monohydrate (COM), struvite, uric acid, and cystine, with high comminution rates.

#### Dose settings and size of stone fragments

Maxwell et al. highlighted that higher ultrasound frequencies (800 kHz) produced smaller fragments (< 1 mm), while lower frequencies (170 kHz) resulted in larger fragments (3–4 mm), allowing for controlled fragmentation tailored to clinical needs [6]. Simulations demonstrate that increasing pressure amplitude (up to 6.5 MPa) and ultrasound frequency (up to 800 kHz), improved stone fragmentation while minimizing tissue damage, potentially making it safer and more effective than shock wave lithotripsy (SWL). Moghimnezhad et al. [11] which was similarly shown by Bailey et al. [12] especially effective for small stones (1–3 mm), achieving 87% mass reduction to submillimeter dust within 10 min. Larger stones (3–5 mm) fragmented effectively at both low (390 kHz) and high frequencies, implying that a mixed-frequency approach show potential benefits. Ramesh et al. [13] confirmed BWL’s broad applicability by fragmenting 89% of human urinary stones within 30 min and validated the use of ultrasonic propulsion [14] for real-time assessment of fragmentation.

#### Physical mechanisms of burst wave

Sapozhnikov et al. [15] developed a theoretical model indicating that stress amplification within stones depends on ultrasound frequency and stone geometry and identified optimal frequency ranges (*ka* ≈ 2–5) for different stone compositions, supporting a sequential fragmentation strategy using low and high frequencies. Maxwell et al. [16, 17] further investigated the physical mechanisms of BWL, using photoelastic imaging to confirm that elastic waves (shear and longitudinal) propagate within the stone, forming standing waves that create localized stress points and initiate fractures. The results demonstrated that lower frequencies produced larger fragments, whereas higher frequencies generated smaller, passable fragments.

### What we learned from pre-clinical experiments

#### Stone comminution

Wang et al. evaluated the effectiveness and safety of BWL, using three porcine models implanted with 6–7 mm human COM kidney stones [18]. Stones were targeted and treated transcutaneously using a BWL and an ultrasound imaging probe for guidance with an exposure time of three 10-minute intervals per stone. Findings showed at least 50% of each stone was reduced to < 2 mm fragments and 100% of four stones were reduced to < 4 mm fragments. Yet, in three out of five treatments, stones were completely disintegrated.

Maxwell et al. [19] developed a BWL system tailored for treating obstructing ureteric stones in pet cats using 35 natural COM stones. The system could effectively fragment stones into pieces smaller than 1 mm—with an average reduction of stone mass by 73–97% within 30 to 50 min of treatment. Overall, the study supports the potential of this adapted BWL system as it holds the promise of effective stone fragmentation and minimal associated tissue damage.

Holmes et al. [20] applied BWL combined with ultrasonic propulsion to treat urolithiasis in a bottlenose dolphin and a harbor seal. In the dolphin, two BWL treatments fragmented ureteral stones, and the ultrasonic propulsion helped reposition the fragments, demonstrating that BWL and ultrasonic propulsion combined can effectively address urinary stones in animals with challenging anatomies.

#### Tissue injury

Regarding safety, Wang et al. showed no skin or intervening tissue damage, a minimal injury to the functional renal volume on magnetic resonance imaging (MRI) and minor petechial hemorrhages in the urothelium near the stone fragments with no significant renal parenchymal injury on histological assessment [18, 21]. Connors et al. [22] investigated for immediate functional or morphological kidney damage in 12 female pigs split into two groups: one group received BWL treatment (18,000 pulses delivered at 10 pulses per second with 20 cycles per pulse, using specific pressure settings of 12 MPa positive and − 7 MPa negative), while the other served as sham controls. The results showed no significant changes in renal function, nor did it produce measurable hemorrhagic lesions compared to sham-treated kidneys. The authors suggest that the lower pressure and reduced cavitation associated with BWL may account for its tissue-sparing effects.

Shelton et al. [23] evaluated if BWL causes renal hemorrhage in a therapeutically anticoagulated state using a porcine model with unilateral heparinized kidney and contralateral kidney as control. Urine analyses revealed only minimal microscopic hematuria in both groups, with no significant differences. MRI-based lesion analysis showed that the hemorrhagic injury in BWL-treated kidneys was not different from controls. This suggests that BWL can safely fragment kidney stones without inducing significant bleeding, even in an anticoagulated state, offering a promising alternative for patients who require continuous anticoagulation.

May et al. [24] investigated whether BWL induces detectable renal injury by real time monitoring of porcine kidneys using custom BWL transducers at 170 kHz and 335 kHz. Most lesions induced by BWL, especially those generated using the 335 kHz transducer, were very small and insignificant but larger injuries were observed at 170 kHz. Histologically, the injuries were like those seen with SWL, characterized by focal tubular damage and intraparenchymal hemorrhage. These results suggest that real-time ultrasound monitoring, combined with MRI-based quantification, offers an effective framework for preclinical safety evaluation of BWL, potentially allowing clinicians to adjust treatment parameters in real time to minimize renal injury.

### What we learned from human studies

To date, clinical studies have demonstrated BWL effectiveness in stone comminution and stone displacement by combining with ultrasonic propulsion, as well as safety and tolerability in awake patients within a short ten minutes treatment.

Harper et al. reported the first account of in-human BWL trial on kidney and ureteral stone in 2021 [25]. Effectiveness and safety of in-human BWL were demonstrated by the complete comminution of a 7 mm renal stone into < 2 mm fragments on endoscopic view within 10 min (8.5 min at 7 MPa peak negative pressure and 10 Hz repetition rate, and 30 s at 6 MPa and 17 Hz) in the first patient under anaesthesia. The BWL pulses were triggered by the operator during phases of the breathing cycle when the stone falls within the target zone on real-time ultrasound. Tolerability of in-human BWL was demonstrated by the first awake patient with 7.5 mm symptomatic ureterovesical stone reporting no added discomfort from BWL treatment. The ability to control breathing in an awake patient might improve the treatment outcomes.

Hall et al. evaluated the efficacy and usage of ultrasonic propulsion alone or with BWL bursts in 24 unanaesthetised patients with ureteral stones prospectively [26]. The primary outcome of stone displacement occurred in 66%; whilst secondary outcome was a 86% distal ureteral stone passage rate. The pain scores were significantly lowered during the procedure from 2.1±2.3 to 1.6±2.0 (*p* = 0.03). The study pointed out room for finetuning of transducer engineering when 6 out of 26 patients failed screening due to a large skin to stone distance.

Harper et al. looked into the effectiveness of stone fragmentation in 19 patients with BWL in ten minutes [7], where they reported a median stone comminution of 90% stone volume in 91% targeted stones, of which 39% targeted stones were completely fragmented (defined as all fragments = < 2 mm). The study examined for any tissue injury by independent blinded visual scoring of ureteroscopic video, where only mild petechiae and small blood clots were noted with no reported adverse events, confirming its safety.

Chew et al. [8] reported the first in-human multi-center prospective trial of BWL on urinary tract stones with therapy dose levels between 4.5 and 8 MPa of acoustic negative pressure (NCT03811171). BWL successfully fragmented stones in 88% of cases, with 51% achieving residual fragments of less than or equal to 2 mm on non-contrast CT scan at 10 ±2 weeks. 86% of trial patients underwent BWL without sedation in a clinic setting over 30 min. The procedure was well-tolerated, with only Clavien-Dindo Grade 1 adverse events, most commonly transient hematuria (93.2%) followed by renal colic treated with analgesics (56.8%). Despite being able to treat stones across depths ranging from 4 cm to 14 cm with three different treatment probes, limitations exist including challenges in treating certain stone locations due to ultrasound constraints. As BWL is an ultrasound based technology, stone located at proximal, mid ureter and some upper pole calyceal stones are not amenable to BWL if not identified by ultrasound, particularly if obscured by air or overlying bone.

### Ongoing trials of BWL

Ongoing trials of BWL focus on two main themes, namely consolidation of the efficacy and safety of BWL, and improving stone-free rates by combining BWL with other technologies.

There are currently two ongoing studies registered in the ClinicalTrials.gov. Chew et al. showed the feasibility of BWL in 44 patients with upper urinary tract stones [8], paving ground work for the pivotal trial. SonoMotion (San Mateo, California) designed a trial to include patients to further evaluate the efficacy and safety of BWL (NCT05701098): SOUND Pivotal Trial– Sonomotion stOne comminUtion resoNance ultrasounD. This prospective, open-label multicenter, single-arm study, plans to include up to 116 patients with upper urinary tract stones of 4–10 mm. The primary outcome is to investigate the safety (in terms of adverse events, unplanned emergency department or clinic visits, and the need for further intervention) and effectiveness (size of fragment at 10 weeks after treatment) of BWL. This study is currently recruiting at 10 study locations, all located in North America.

The other clinical trial registered is to study the combination of BWL with ultrasound propulsion. The previous study published by Hall et al. in 2022 only included 13 patients who used both technologies, and the primary outcome was stone motion [26]. The registered trial by the University of Washington is titled Ultrasound to Facilitate Stone Passage (NCT04796792, or Propulse2 [27]). It is a prospective, open-label, multicenter study to determine whether the combination of breaking and repositioning of stones with ultrasound under no anesthesia can result in better stone passage. The trial has three phases: phase 1 is to include 20 subjects that tests for the feasibility; phase 2a is a randomized control trial which aims to include a total of 100 subjects, in a 1:1 ratio; phase 2b is a feasibility study of BWL by 20 subjects with spinal cord injury. Phase 2 of this study will be conducted concurrently after phase 1 results are reviewed by the FDA and approved to proceed to phase 2. This study is not yet recruiting.

### Take home messages and future directions

Animal studies, reinforced by experimental evidence and substantial provisional findings replicated in human trials, demonstrate BWL as a promising non-invasive alternative to SWL. BWL offers controllable stone fragmentation and the capability to generate dust by modulating shock wave frequency. This coupled with minimal tissue damage and nephron injury is a significant leap forward from SWL. Furthermore, BWL’s stone propulsion feature may enhance treatment efficacy for lower pole and ureteric stones. When combined with its portable design, these benefits position BWL as a potential candidate for portable office-based or even home-based lithotripsy, marking a transformative step in urological care. Although BWL still faces the same challenges as SWL regarding ultrasound constraints with acoustic window and skin-to-stone distance, these limitations will hopefully be resolved in the future as technology advances. Finally, BWL’s safety profile in anticoagulated patients without the need of anesthesia could establish it as a groundbreaking advancement in urolithiasis management. The potential of BWL is yet unrealized. Its utility as an alternative to SWL or as an adjunct to ureteroscopy in relocation and/or fragment disintegration can be defined once it is commercially available. Its portability best suits stones and patients amenable for ambulatory procedures.

## Conclusion

BWL represents a paradigm shift in lithotripsy, offering controlled fragmentation, reduced tissue injury, fragment propulsion and portability for office-based or ambulatory care. Early clinical evidence underscores its safety and efficacy, even in anticoagulated patients. While large-scale trials are needed to solidify its role, BWL’s procedural flexibility positions it as a transformative alternative to SWL, poised to redefine urolithiasis management.

## Electronic supplementary material

Below is the link to the electronic supplementary material.

Supplementary Material 1

## Data Availability

No datasets were generated or analysed during the current study.
