# Supplementary figures and images for: Burst wave lithotripsy - a paradigm shift: inferences from a scoping review
Source: World J Urol. 2025 Apr 25;43(1):250. doi: 10.1007/s00345-025-05645-x (PMC12031800; doi:10.1007/s00345-025-05645-x)

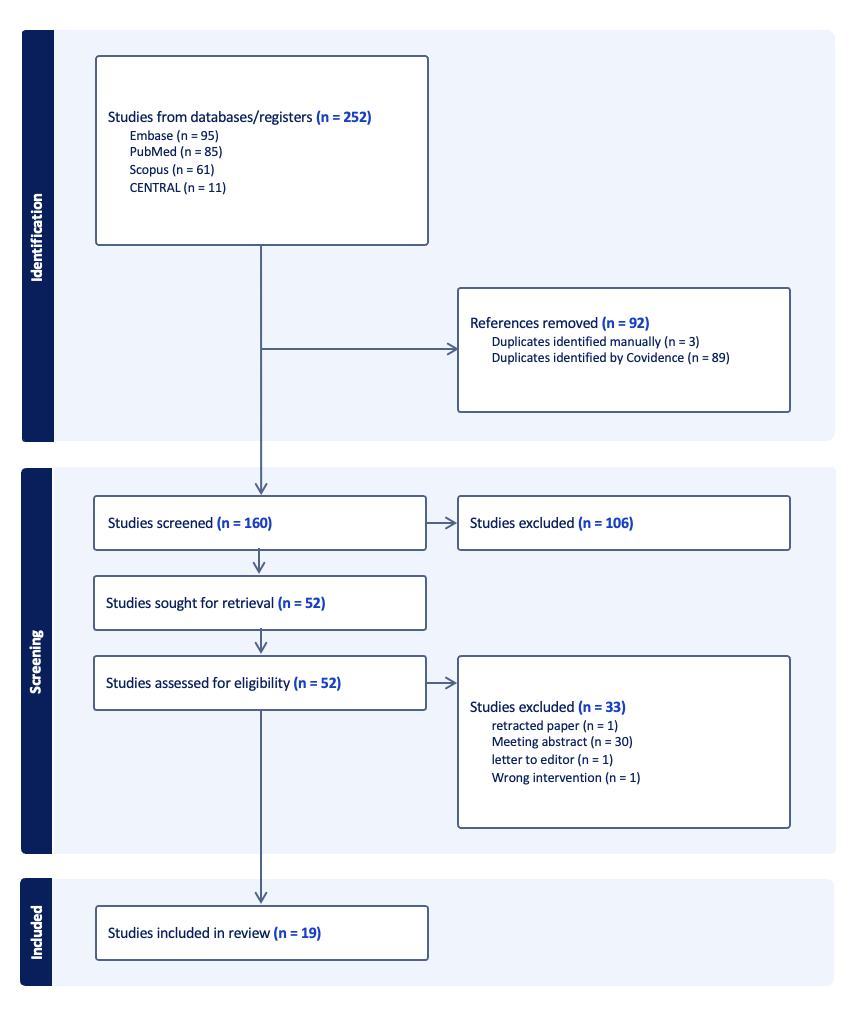


Supplementary Figure. PRISMA Flowchart.

Supplement: Supplementary file 1 — Supplementary Material 1 [file 345_2025_5645_MOESM1_ESM.docx]
